# Living with diabetes and its impact on mental health: results of an online survey

**DOI:** 10.1097/XCE.0000000000000262

**Published:** 2022-03-29

**Authors:** Mike Stedman, Saydah Eltom, Emma Solomon, Rustam Rea, Katherine Grady, Nadia Chaudhury, Stephen Brown, Angela Paisley, Roger Gadsby, Adrian H. Heald

**Affiliations:** a Medical Research, Res Consortium, Andover; Departments of b Diabetes and Endocrinology; c Clinical Psychology, Salford Royal Hospital, Salford; d Oxford Centre for Diabetes, Endocrinology and Metabolism, University of Oxford, Oxford; e Research for the Future, Northern Care Alliance National Health Service Group, Salford; f University Hospitals Coventry and Warwickshire, Coventry; g The School of Medicine and Manchester Academic Health Sciences Centre, University of Manchester, Manchester; h University of Warwick Medical School, Coventry, UK

People with diabetes often experience low mood. There is considerable evidence for diabetes reducing the quality of life (QOL) and mental health [1–3]. The underlying factors that might contribute to this are less well understood and were explored in this recent study.

We conducted an online survey of people with diabetes, who had agreed to be involved through the research for the future (RfTF) project, in relation to their lived experience of the condition [4]. PHQ-9 (depression) [5], Diabetes Distress Screening Scale (DDSS) [6] and EQ5D5L QOL [7] questionnaires were completed by 130 people with diabetes and their clinical records were also examined. The aim of the study was to determine the prevalence of low mood and reported distress in people with diabetes.

Ethical approval was obtained from the Greater Manchester West Research Ethics Committee: REC reference: 20/LO/0738 specifically citing RfTF as a recruiting ‘venue’. RfTF is an National Health Service-supported organization that encourages people to become more involved with health research in their local area. The RfTF database as an National Institute for Health Research resource is deemed to be broadly representative of people with diabetes living in England.

Of the 130 people who responded (22% response rate), 45 had type 1 diabetes and 85 had type 2 diabetes (T2DM). A total of 56% were women and 44% were men. The majority of participants were under primary care. The median age was 59 [interquartlie range (IQR), 47–67] years. Overall median scores were: EQ5D5L 0.74 (IQR, 0.64–0.85) (lower than the UK population median score of 82.8), DDSS 1.9 (IQR, 1.3–2.7) (≥2 indicates moderate distress) and PHQ-9 5 (IQR, 2–11) (≥5 indicates depression).

Worse scores reflecting higher diabetes distress (DDSS), lower QOL EQ5D5L and higher depression (PHQ-9) were linked to female sex, younger age, less years after initial diagnosis and obesity.

The 30% of people with a history of prescribed antidepressant medication in the previous 12 months also showed worse scores (47% higher than those with no antidepressant use history). The DDSS score elevation came from increases in emotional burden and regimen related distress. Score variances were not linked to diabetes type, prescription of insulin or the change in blood glucose control over the last three HbA1c measurements.

Clinically significant depression has been reported in up to one of every four people with T2DM [8]. The results of our study should be placed in the context of this and similar observations. We accept that our sample was self-selected so any findings should be treated with caution. However, we feel that the greater impact of diabetes on mental health apparent in younger women and in people with a shorter duration of diabetes and those with a BMI of at least 30 are relevant.

We suggest that these factors be considered when planning psychosocial interventions and behavior change messaging to support people with diabetes, in relation to the multiple challenges that they face, particularly given the impact of the COVID-19 pandemic on routine care for people with diabetes in the UK and elsewhere [4].

## Acknowledgements

### Conflicts of interest

There are no conflicts of interest.
